# Associations of childhood economic and psychosocial conditions with later-life cognitive function: a longitudinal analysis of the China health and retirement longitudinal study (2011–2020)

**DOI:** 10.1186/s12889-026-26826-2

**Published:** 2026-02-27

**Authors:** Tung Le, James Gilleen, Amanda Lee, Asri Maharani

**Affiliations:** 1 Mental Health Research Group, Division of Nursing, Midwifery and Social Work, Faculty of Biology, Medicine and Health, School of Health Sciences, The University of Manchester, Manchester, M13 9PL UK; 2 Faculty of Health and Education, School of Nursing and Public Health, Manchester Metropolitan University, Manchester, M15 6BX UK; 3 General Planning Department, Hanoi Medical University Hospital, Hanoi Medical University, Hanoi, Vietnam

**Keywords:** Cognitive function, Childhood socioeconomic status, Life course, Longitudinal study, China, CHARLS, Health disparities

## Abstract

**Background:**

Cognitive decline is a significant public health challenge in China. Childhood socioeconomic status has a lasting impact on later-life health, but the specific life-course pathways that link early-life adversity to later-life cognitive function remain unclear. This study aimed to examine the association between childhood economic and psychosocial conditions and later-life cognitive function in older Chinese adults, and additionally, whether social and health factors in adulthood influenced or accounted for this association.

**Methods:**

We analysed 10,950 participants from all waves of the China Health and Retirement Longitudinal Study (2011–2020). Early-life exposures were childhood economic indicators (parental occupation and perceived family financial status) and psychosocial indicators (childhood loneliness and parental relationships). Cognitive function was the dependent variable. We utilised multilevel growth curve models with random intercepts, random slopes, and quadratic age terms to estimate cognitive trajectories. A sequential modelling strategy evaluated whether adult social factors and health factors accounted for observed associations.

**Results:**

The sample comprised 10,950 individuals with a mean age of 58.22 ± 8.90 years. In models adjusted only for age and sex, poorer childhood financial status was associated with lower cognitive function. However, after accounting for adult social and health factors, this association was substantially attenuated (reduced by 42%), though it remained statistically significant (β = -0.16, *p* = 0.023). Similarly, the protective effects of several early-life indicators were partially explained by adult-life pathways. In particular, the association for better childhood financial status was fully attenuated after adjustment. Nevertheless, having a father in non-agricultural employment (β = 0.74, *p* < 0.001) and not experiencing childhood loneliness (β = 0.33, *p* < 0.001) remained robustly associated with higher cognitive performance even in the fully adjusted model. Among adult factors, higher educational attainment (β = 3.80, *p* < 0.001), fewer Instrumental Activities of Daily Living limitations (β = -0.35, *p* < 0.001), and lower depressive symptom scores (β = -0.08, *p* < 0.001) were strongly associated with cognitive function.

**Conclusions:**

Childhood economic and psychosocial disadvantages have persistent, long-term effects on cognitive functioning in later life. These effects are substantially explained by adult life pathways, particularly educational attainment, physical frailty, and mental health. These findings suggest that interventions targeting both childhood adversity and adult-life risk factors may be effective in mitigating cognitive decline.

**Supplementary Information:**

The online version contains supplementary material available at 10.1186/s12889-026-26826-2.

## Introduction

Good cognitive functional status is key to maintaining independence in older adults, yet cognitive decline poses a significant public health challenge. As the ageing population increases, cognitive decline will pressure healthcare systems worldwide [1]. Globally, almost 60 million people currently live with dementia, a figure projected to rise to over 152 million by 2050 [2]. This burden is particularly acute in China, which has the world’s largest ageing population and is projected to have 23.3 million individuals with dementia by 2030 [3]. Understanding the modifiable factors that influence cognitive trajectories is, therefore, an urgent public health priority [4].

Childhood socioeconomic status (SES) is a well-established early-life factor with a lasting impact on health in later life [5], often referred to as the “long arm of childhood” [6]. This association is a central concept within the life-course framework [7], which is explained by two key mechanisms. First, the cumulative disadvantage hypothesis suggests that early disparities in family income, parental occupation, and education can limit access to resources and accumulate over time [8, 9], thereby undermining cognitive resilience [10]. Second, cognitive reserve theory suggests that enriched childhood environments, linked to higher SES and strong social networks, build cognitive reserve that mitigates decline [11–13]. Evidence consistently links childhood disadvantage to an increased risk of poorer cognitive outcomes in later life [11, 14].

Additionally, childhood disadvantage extends beyond economic constraints to include psychosocial dimensions such as loneliness, peer relationships, and parent-child bonds, which may independently influence cognitive ageing [15–17]. Previous studies confirm that adverse psychosocial experiences in childhood, including isolation and poor-quality relationships, are associated with an increased risk of cognitive decline and dementia, even after accounting for socioeconomic status [16, 17]. Recent studies in China have also specifically linked deficits in childhood peer relationships [18] and poorer parent-child bonds [19] to worse later-life cognitive outcomes.

Despite this evidence, childhood economic and psychosocial conditions are typically examined in isolation. Economic conditions are operationalised via indicators like parental occupation and wealth [16, 17], while psychosocial conditions are measured via self-reported loneliness or relationship quality [19]. This separation is a key limitation, as material and psychosocial environments are closely interrelated and may influence cognitive ageing through distinct but overlapping pathways. Economic hardship can constrain psychosocial and cognitive development [20], while strong psychosocial bonds may buffer the impact of financial adversity [21]. Furthermore, they align with divergent theoretical pathways. Economic conditions are theorised to shape cognitive reserve through material investment in education and nutrition [11], whereas psychosocial conditions are posited to build cognitive resilience through psychosocial mechanisms like secure attachment [20]. Therefore, a combined framework is essential to test their independent and interdependent relationships, a key gap in current life course research.

In life-course research, adult factors are typically positioned within two theoretical frameworks, including the pathway (mediation) model and the buffering (moderation) model [22]. Prior studies often treat adult social factors, such as educational attainment and occupational class, as critical mediators that transmit early-life disadvantage into later life by shaping cognitive reserve [23]. Furthermore, some research suggests that adult SES may act as a moderator. For example, Wang et al. (2022)’s cross-sectional evidence indicates that high adult socioeconomic status can modestly buffer the impact of childhood poverty [24]. Similarly, longitudinal research using the Health and Retirement Study indicates that adult health factors, including depression, chronic conditions, and functional limitations, are positioned as proximal drivers of decline [25]. These are often “chained” to early adversity through the biological embedding of stress, which accelerates neurostructural ageing and impairs brain maintenance.

Building on the need for an integrated framework, other methodological and conceptual gaps persist. First, although the influence of adult factors is well understood, the specific interplay between early-life disadvantage and adult-life pathways remains underexplored. Second, many previous studies have not used the advanced multilevel growth curve models, which estimate individual-specific intercepts and slopes, account for within-person correlation, handle unbalanced and irregular follow-up and missing data under missing at random, and incorporate time-varying covariates, capabilities not offered by change scores, repeated measures ANOVA, or generalised estimating equations (26).

To address existing conceptual and methodological gaps, this study applies a life-course approach using five waves of data (2011–2020) from the China Health and Retirement Longitudinal Study (CHARLS). The childhoods of CHARLS participants spanned several transformative and tumultuous periods in modern Chinese history, including the Anti-Japanese War (1937–1945), the Civil War (1927–1949), the Mao era (1949–1976), and the early Reform period beginning in 1978 [26–29]. Before 1949, the socioeconomic landscape was characterised by a predominantly agrarian society with deep-seated feudal structures, where early-life adversity was profoundly shaped by persistent warfare and extreme rural poverty [30]. This era encompassed a transition from this traditional structure to a state-organised society and eventually to market-oriented economic reforms [26–28]. Such a distinctive historical and social context provides a unique opportunity to examine how structural disadvantages and social conditions, substantially different from Western class systems [31–33], affect long-term cognitive health.

Within this context, our study provides an integrative analysis by examining both economic factors (family finances, parental occupation) and psychosocial factors (parental relationships, loneliness) simultaneously, rather than in isolation as in previous research. To address the complexities of non-linear decline and high attrition, we apply robust multilevel growth curve models [34] with random intercepts, random slopes, and quadratic age terms. This approach allows us to disentangle baseline differences from rates of cognitive change and use a sequential modelling approach to identify which adult social and health pathways most strongly explain these early-life associations.

We hypothesise that: (H1) Higher childhood economic and better childhood psychosocial factors are associated with a higher level of cognitive function in later life; (H2) Social and health factors in adulthood, including educational attainment and health status, are expected to partially explain the associations between childhood economic and psychosocial conditions and later-life cognition.

## Methods

### Data and study sample

Participants for this study were drawn from waves 1 (2011) to 5 (2020) of the CHARLS, a nationally representative survey examining the health, economic, and social conditions of China’s population aged 45 and older [35]. To construct the longitudinal dataset spanning all five waves, we merged three separate CHARLS data releases: the harmonised dataset (which includes Waves 1–4), the Wave 5 dataset (2020), and the Life History dataset (administered retrospectively in 2014). The harmonised and Wave 5 datasets together provide five waves of repeated measures from 2011 to 2020. The Life History dataset contributes time-invariant childhood socioeconomic and psychosocial relationship variables collected once in 2014. We included respondents who were aged 45 years or older at Wave 1, participated in the first wave, and had completed the Life History module in 2014. Because the Life History module was administered only in 2014, individuals who joined CHARLS after 2014 were not included. Childhood measures are drawn from the 2014 assessment and treated as fixed baseline characteristics. Cognitive function was assessed in each wave; all participants had cognitive data in at least one wave between Waves 1 and 5. The final baseline sample comprised 10,950 individuals with childhood data and at least one cognitive assessment.

This study used anonymised data from the CHARLS. Ethical approval for all CHARLS waves was granted by the Institutional Review Board at Peking University (IRB00001052-11015). All participants provided informed consent. The CHARLS dataset is publicly available to researchers through the official website [36].

## Measures

### Cognitive function

The dependent variable in this study was cognitive function, measured as the repeatedly assessed total cognitive score across all waves. Cognitive function was measured using an adapted Chinese version of the Mini-Mental State Examination [37], encompassing four dimensions: orientation (0–4), verbal episodic memory (immediate and delayed word recall, 0–20), numerical calculation (0–5), and visuoconstruction (figure-copying task scored 1 for an accurate copy and 0 otherwise, 0–1). The total cognitive score (range 0–30) was the sum of the four individual scores, with higher scores indicating better cognitive function.

### Childhood economic indicators

Childhood economic indicators were derived from two items in the 2014 Life History Survey referring to the period before age 17. Family financial situation was assessed with the question, “When you were a child before age 17, compared to the average family in the same community or village at that time, how was your family’s financial situation?” The response options were a lot better off than them, somewhat better off than them, same as them, somewhat worse off than them, and a lot worse off than them. We recoded these as “better” (a lot better or somewhat better), “same”, and “worse” (somewhat worse or a lot worse). Parental occupation was assessed with separate items asking, “What was your female guardian’s usual occupation when you were growing up before you were 17?” and “What was your male guardian’s usual occupation when you were growing up before you were 17?” with response options farming or non-agricultural. We recoded occupations as farming or non-agricultural work.

### Childhood psychosocial indicators

We also assessed two psychosocial indicators from the 2014 Life History Survey. Self-rated childhood loneliness was derived from the question “When you were a child, how often did you feel lonely for not having friends?” with response options often, sometimes, not very often, and never. We recoded often or sometimes as “yes” and not very often or never as “no”. Parental relationships were assessed separately for the mother and for the father using the items “How would you rate your relationship with your mother when you were growing up?” and “How would you rate your relationship with your father when you were growing up?” with response options excellent, very good, good, fair, and poor. We grouped excellent or very good as “very good or excellent”, good as “good”, and fair or poor as “fair or poor”.

### Adult-life social and health factors

We also assessed several adult-life factors that could explain the association between childhood indicators and cognition. These were grouped into two categories. Adult social factors included the highest educational attainment by adulthood (elementary or lower, secondary, high school or higher), marital status (married or partnered, living alone), and living area (rural, urban). Education and marital status were selected as key markers of adult social and economic standing. Educational attainment is a well-established proxy for cognitive reserve and a critical pathway through which early-life advantages may translate into better later-life cognitive health [38]. Marital status, particularly being partnered, often provides ongoing social engagement, emotional support, and economic co-regulation, all of which are protective for cognitive ageing [39]. Adult health and behaviour factors included smoking history (yes, never), participation in any listed social activities in the past month (meeting friends, playing mahjong, chess or cards, going to a community club, helping non-cohabiting family, friends or neighbours, attending a sport, social or other club, taking part in a community organisation, doing voluntary or charity work, caring for a sick or disabled adult who does not live with the respondent, or attending an educational or training course; coded yes or no), chronic diseases (doctor-diagnosed, ever: hypertension, diabetes, cancer, lung disease, stroke, psychiatric disorders, arthritis, and asthma), need for help with Activities of Daily Living (ADL include bathing, dressing, toileting, transferring, continence, and eating; summed as the total number of ADL activities requiring help), Instrumental Activities of Daily Living (IADL include shopping, meal preparation, housekeeping, telephone use, medication management, and money management; summed as the total number of IADL activities requiring help), and depressive symptoms (10-item Center for Epidemiologic Studies Depression Scale, CESD-10 scores, continuous). Adult SES and health variables were measured concurrently with cognitive function in each wave (Waves 1–5) and were treated as time‑varying covariates in longitudinal models.

### Confounders

All models were adjusted for two potential confounders, namely sex (with female as the reference) and age (continuous, time-varying).

### Statistical analysis

**Fig. 1:**
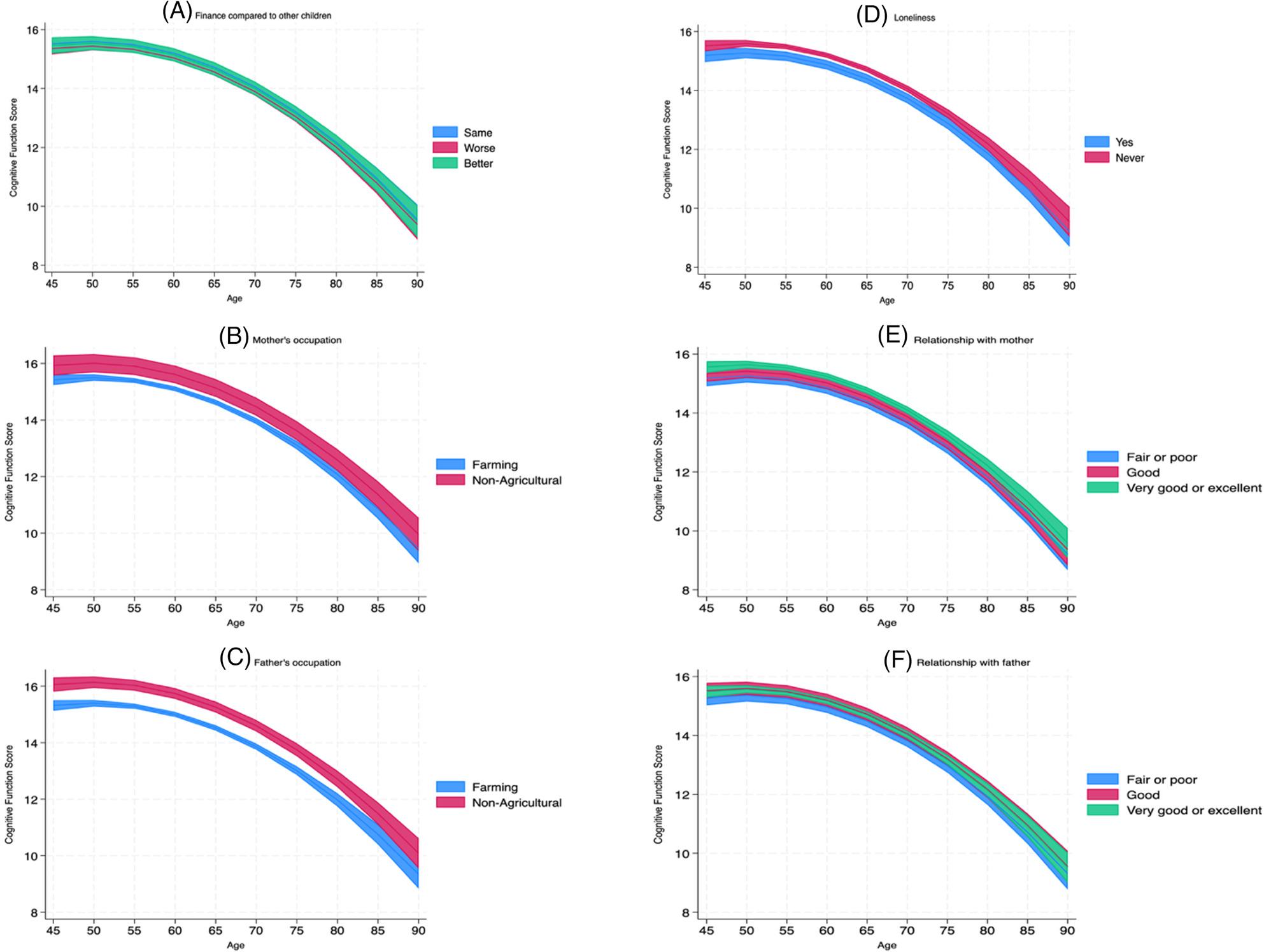
Predicted cognitive trajectories from the full multilevel model (Model 3). * Note: Predicted cognitive trajectories were estimated from Model 3 in Table 2. Each panel displays adjusted predictions across age while holding all other covariates in Model 3 at their means (for continuous variables) or reference levels (for categorical variables). Model 3 simultaneously adjusted for age, sex, educational attainment, marital status, Instrumental Activities of Daily Living (IADL) limitations, depressive symptoms, and all other childhood variables shown in the figure. Shaded areas represent 95% confidenceintervals. **A** Finance compared to other children; **B** Mother’s occupation (**C**) Father’s occupation; **D** Loneliness; **E** Relationship with mother; **F** Relationship with father

Subject characteristics at baseline were summarised with means and standard deviations (SDs) for continuous variables and percentages for categorical variables, with comparisons by sex using Kruskal-Wallis and Chi-square tests, respectively. To test our hypotheses, we employed a sequential modelling approach (also known as stepwise adjustment) to examine the influence of adult-life factors on the childhood associations. The specific statistical tool used for this approach was the multilevel growth curve model, as it is ideal for longitudinal data with repeated measures (Level 1) nested within individuals (Level 2) [40, 41]. Our models included both a random intercept for each individual and a random slope for time (wave, coded 0–4 corresponding to waves 1–5), allowing the expected cognitive score at Wave 1 and the rate of change to vary across participants. To capture the non-linear trajectory of cognitive decline, all models included both linear and quadratic terms for time-varying age. The inclusion of a quadratic age term is a methodological necessity in ageing research, as cognitive decline typically accelerates with increasing age rather than progressing at a constant linear rate [42]. By accounting for this curvilinear trajectory, we ensure that our estimates for the cognitive intercepts and slopes are not biased by an oversimplified linear assumption, thereby more accurately reflecting the biological reality of cognitive ageing. The sequential approach involved building three nested models. Model 1 (baseline effects) tested H1 by estimating the associations of all childhood economic and psychosocial factors with cognitive function, controlling only for confounders (sex, linear and quadratic age) and the random effects. Model 2 then included the adult social factors (education, marital status, and living area) into Model (1) Finally, Model 3 (the full model) added the adult health and behaviour factors (smoking, chronic diseases, ADL, IADL, CESD-10) to Model (2) To test H2, we assessed the extent to which these adult factors influenced the observed associations by examining the attenuation (i.e., reduction in coefficient size and statistical significance) of the childhood factor coefficients from Model 1 to Model (3) We also compared the relative fit of these nested models using the Akaike Information Criterion (AIC) and the Bayesian Information Criterion (BIC), with lower values indicating a better model fit. Finally, predicted trajectories for Fig. 1 were generated from the fully specified growth curve model (Model 3). 95% confidence intervals are plotted around the trajectories. To address the cognitive reserve hypothesis specifically, we also tested interaction terms between childhood factors and age-related trajectories (using both linear and quadratic age terms) to evaluate their impact on the rate of cognitive change. The interaction terms between childhood conditions and age-related trajectories were not statistically significant, suggesting that childhood conditions were not associated with the rate of cognitive change, but rather with differences in baseline cognitive level (intercept) (Supplementary Table 2).

To ensure robustness against missing data, we conducted a sensitivity analysis using multiple imputation by chained equations (MICE), following the framework described by White and colleagues [43]. The imputation model employed linear regression for continuous variables and logistic regression for binary variables. The multilevel growth-curve models were then re-estimated using these imputed data to examine whether the observed associations remained consistent. Additionally, to evaluate potential attrition bias, we performed a further sensitivity analysis by re-estimating our final model (Model 3) on the subset of participants who provided complete data for all five waves. All analyses were conducted using Stata version 19.

## Results

Table 1 presents the demographic characteristics of 10,950 participants at baseline, segmented by sex. At baseline, males had a slightly higher mean cognitive score (15.54) than females (14.42, *p* < 0.001), though this variable had 26.80% missing data, which is addressed in our methods. Significant differences were found in early life indicators. Females were more likely to report better childhood financial conditions than males (10.01% vs. 7.77%, *p* < 0.001). Most participants came from agricultural backgrounds, with 94.13% of mothers and 84.15% of fathers working in farming. Childhood loneliness was more prevalent among males (21.19% vs. 19.39% for females, *p* = 0.019). Parent-child relationships also differed, with 22.76% of males reporting “Fair or poor” relationships with their fathers compared to 18.14% of females (*p* < 0.001). The mean age of participants was 58.22 years, with males being slightly older (58.51 years) than females (57.95 years, *p* < 0.001). Educational attainment revealed stark contrasts, with only 1.76% of females achieving high school or higher education compared to 5.44% of males (*p* < 0.001). Most participants were married or in a partnership (89.23%), but females were more likely to live alone (13.39% vs. 7.88% for males, *p* < 0.001). Health variables also displayed significant sex differences. Females had a higher prevalence of hypertension (26.55% vs. 23.53% for males, *p* < 0.001) and arthritis (38.48% vs. 29.07%, *p* < 0.001). Females also reported greater needs for assistance in daily activities (ADL mean: 0.35 vs. 0.25; IADL mean: 0.47 vs. 0.29, *p* < 0.001) and higher mean CESD-10 scores, indicating more depressive symptoms (9.35 vs. 7.35, *p* < 0.001).

**Table 1:** Baseline characteristics of the study population (*n* = 10,950) by sex

Variable	All (*n* = 10,950)	Females (*n* = 5,673)	Males (*n* = 5,277)	*p*-value	Missing data (%)
Baseline cognitive score, mean (SD)	14.99 (4.61)	14.42 (4.89)	15.54 (4.25)	< 0.001	26.8
Finance compared to other children, n (%)				< 0.001	0
Same	5,766 (52.66)	2,978 (52.49)	2,788 (52.83)		
Worse	4,206 (38.41)	2,127 (37.49)	2,079 (39.40)		
Better	978 (8.93)	568 (10.01)	410 (7.77)		
Mother’s occupation, n (%)				0.753	0
Farming	10,307 (94.13)	5,336 (94.06)	4,971 (94.20)		
Non-Agricultural	643 (5.87)	337 (5.94)	306 (5.80)		
Father’s occupation, n (%)				0.055	0
Farming	9,214 (84.15)	4,737 (83.50)	4,477 (84.84)		
Non-Agricultural	1,736 (15.85)	936 (16.50)	800 (15.16)		
Childhood loneliness, n (%)				0.019	0
Yes	2,218 (20.26)	1,100 (19.39)	1,118 (21.19)		
Never	8,732 (79.74)	4,573 (80.61)	4,159 (78.81)		
Relationship with mother, n (%)				< 0.001	0
Fair or poor	1,978 (18.06)	945 (16.66)	1,033 (19.58)		
Good	1,977 (18.05)	1,066 (18.79)	911 (17.26)		
Very good or excellent	6,995 (63.88)	3,662 (64.55)	3,333 (63.16)		
Relationship with father, n (%)				< 0.001	0
Fair or poor	2,230 (20.37)	1,029 (18.14)	1,201 (22.76)		
Good	2,060 (18.81)	1,114 (19.64)	946 (17.93)		
Very good or excellent	6,660 (60.82)	3,530 (62.22)	3,130 (59.31)		
Age, mean (SD)	58.22 (8.90)	57.95 (8.98)	58.51 (8.80)	< 0.001	0
Education, n (%)				< 0.001	0
Elementary or lower	7,346 (67.09)	4,369 (77.01)	2,977 (56.41)		
Secondary education	3,217 (29.38)	1,204 (21.22)	2,013 (38.15)		
High school or higher	387 (3.53)	100 (1.76)	287 (5.44)		
Marital status, n (%)				< 0.001	0.04
Living alone	1,175 (10.73)	759 (13.39)	416 (7.88)		
Married or partnered	9,771 (89.23)	4,911 (86.61)	4,860 (92.12)		
Living area, n (%)				< 0.001	0.12
Urban	1,881 (17.18)	891 (15.73)	990 (18.78)		
Rural	9,056 (82.70)	4,774 (84.27)	4,282 (81.22)		
Smoking history, n (%)				< 0.001	0.51
Never	6,536 (59.69)	5,212 (92.43)	1,324 (25.19)		
Yes	4,359 (39.81)	427 (7.57)	3,932 (74.81)		
Social activity attendance, n (%)				0.617	5.87
No	5,563 (50.80)	2,945 (54.21)	2,618 (53.71)		
Yes	4,744 (43.32)	2,488 (45.79)	2,256 (46.29)		
Hypertension, n (%)	2,720 (24.84)	1,490 (26.55)	1,230 (23.53)	< 0.001	1.01
Diabetes, n (%)	625 (5.71)	351 (6.28)	274 (5.26)	0.023	1.39
Cancer, n (%)	85 (0.78)	52 (0.93)	33 (0.63)	0.083	0.91
Lung Disease, n (%)	994 (9.08)	431 (7.66)	563 (10.75)	< 0.001	0.78
Stroke, n (%)	234 (2.14)	106 (1.88)	128 (2.44)	0.044	0.71
Psychiatric Disorder, n (%)	132 (1.21)	83 (1.48)	49 (0.94)	0.01	0.86
Arthritis, n (%)	3,692 (33.72)	2,168 (38.48)	1,524 (29.07)	< 0.001	0.66
Asthma, n (%)	439 (4.01)	191 (3.40)	248 (4.74)	< 0.001	0.89
ADL needs help, mean (SD)	0.30 (0.89)	0.35 (0.94)	0.25 (0.82)	< 0.001	1.63
IADL needs help, mean (SD)	0.38 (0.95)	0.47 (1.03)	0.29 (0.84)	< 0.001	1
CESD-10 score, mean (SD)	8.41 (6.29)	9.35 (6.56)	7.35 (5.79)	< 0.001	6.68

*Values are presented as mean (SD) for continuous variables and n (%) for categorical variables. P-values indicate comparisons between men and women at the baseline. Continuous variables were compared using the Kruskal-Wallis rank-sum test. Categorical variables were compared using Pearson’s chi-square test of independence

Table 2 displays the results of the three sequential multilevel growth curve models.

**Table 2:** Multilevel growth curve models of childhood indicators on cognitive function

Variable	Model 1		Model 2		Model 3	
	β (SE)	*p*-value	β (SE)	*p*-value	β (SE)	*p*-value
Finance compared to other children (ref: Same)						
Worse	-0.48 (0.08)	< 0.001	-0.28 (0.07)	< 0.001	-0.16 (0.07)	0.023
Better	0.37 (0.13)	0.006	-0.02 (0.12)	0.88	-0.06 (0.12)	0.639
Mother’s occupation (ref: Farming)						
Non-Agricultural	1.69 (0.18)	< 0.001	0.67 (0.17)	< 0.001	0.51 (0.17)	0.002
Father’s occupation (ref: Farming)						
Non-Agricultural	1.59 (0.12)	< 0.001	0.85 (0.11)	< 0.001	0.74 (0.11)	< 0.001
Loneliness (ref: Yes)						
Never	0.81 (0.09)	< 0.001	0.52 (0.09)	< 0.001	0.33 (0.09)	< 0.001
Relationship with mother (ref: Fair/poor)						
Good	0.35 (0.17)	0.037	0.21 (0.15)	0.173	0.16 (0.15)	0.294
Very good/excellent	0.63 (0.14)	< 0.001	0.43 (0.13)	0.001	0.38 (0.13)	0.003
Relationship with father (ref: Fair/poor)						
Good	0.34 (0.16)	0.032	0.26 (0.15)	0.07	0.24 (0.14)	0.099
Very good/excellent	0.40 (0.14)	0.003	0.27 (0.13)	0.034	0.20 (0.12)	0.099
Age (linear)	0.27 (0.04)	< 0.001	0.33 (0.04)	< 0.001	0.37 (0.04)	< 0.001
Age (quadratic)	-0.003 (0.000)	< 0.001	-0.003 (0.000)	< 0.001	-0.004 (0.000)	< 0.001
Sex (ref: Female)						
Male	1.47 (0.07)	< 0.001	0.66 (0.07)	< 0.001	0.66 (0.09)	< 0.001
Education (ref: Elementary or lower)						
Secondary			3.12 (0.08)	< 0.001	2.76 (0.08)	< 0.001
High school or higher			4.32 (0.18)	< 0.001	3.80 (0.18)	< 0.001
Marital status (ref: Living alone)						
Married/partnered			0.60 (0.09)	< 0.001	0.44 (0.10)	< 0.001
Living area (ref: Urban)						
Rural			-0.72 (0.08)	< 0.001	-0.82 (0.09)	< 0.001
Smoking (ref: Never)						
Yes					-0.19 (0.09)	0.034
Social activity (ref: No)						
Yes					0.59 (0.05)	< 0.001
Hypertension (ref: No)					0.07 (0.06)	0.267
Diabetes (ref: No)					0.13 (0.10)	0.209
Cancer (ref: No)					0.50 (0.24)	0.034
Lung Disease (ref: No)					0.06 (0.09)	0.531
Stroke (ref: No)					-0.08 (0.15)	0.585
Psychiatric (ref: No)					-0.20 (0.22)	0.373
Arthritis (ref: No)					-0.05 (0.06)	0.387
Asthma (ref: No)					-0.13 (0.14)	0.357
ADL needs help					-0.03 (0.04)	0.477
IADL needs help					-0.35 (0.04)	< 0.001
CESD-10 score					-0.08 (0.00)	< 0.001
Random Effects (SD)						
sd(_cons) [Intercept]	3.12 (0.03)		2.74 (0.03)		2.57 (0.03)	
sd(wave) [Slope]	0.61 (0.02)		0.52 (0.02)		0.60 (0.03)	
sd(Residual)	3.04 (0.02)		3.07 (0.02)		3.05 (0.02)	
Model Fit						
Log likelihood	-94162.42		-91832.13		-80285.03	
AIC	188356.8		183704.3		160636.1	
BIC	188491.7		183872.5		160909.3	

* β (*SE*) Coefficient (Standard Error), *SD * Standard Deviation, *AIC * Akaike Information Criterion, *BIC * Bayesian Information Criterion

Model 1 tested the baseline association between childhood indicators and cognitive function, controlling for age and sex. As hypothesised (H1), both economic and psychosocial indicators showed significant associations with cognitive function in later life. For instance, “Worse” childhood financial status was linked to a 0.48-point lower cognitive score (*p* < 0.001). Conversely, having a non-agricultural father’s occupation (β = 1.59, *p* < 0.001), never feeling lonely (β = 0.81, *p* < 0.001), and having a “Very good or excellent” maternal relationship (β = 0.63, *p* < 0.001) were all associated with significantly higher cognitive scores.

Model 2 introduced adult social factors to test H2. The coefficients for all childhood indicators attenuated, or reduced, confirming that these adult factors explained a portion of the baseline association. For example, the effect of “Worse” childhood financial status was reduced by 42% (β = -0.28, *p* < 0.001). The association for “Better” finance was fully attenuated and lost statistical significance (*p* = 0.880). This suggests that a large portion of the childhood effect is influenced by adult social factors, particularly education, which was the strongest predictor in the model (e.g., High school: β = 4.32, *p* < 0.001).

Model 3 added adult health and behavioural factors to the full model. The childhood coefficients attenuated further. The “Worse” childhood financial status coefficient was reduced again to -0.16 (*p* = 0.023), while the effect of a “Very good or excellent” paternal relationship effect attenuated to no longer being statistically significant (*p* = 0.099). However, “Father’s occupation” (β = 0.74, *p* < 0.001), “Mother’s occupation” (β = 0.51, *p* = 0.002), and “Never lonely” (β = 0.33, *p* < 0.001) all remained significant predictors even in the final model. This suggests that their impact on cognition is only partially explained by adult social and health status. The strongest adult predictors were education, with secondary and high school or higher associated with higher cognitive scores relative to elementary or lower (β = 2.76 and 3.80; both *p* < 0.001). Additionally, IADL needs, where more activities requiring help predicted lower scores (β = -0.35, *p* < 0.001), and depressive symptoms, where higher CESD-10 scores predicted lower cognition (β = -0.08, *p* < 0.001).

The random effects confirm our sequential analysis. The random intercept variance, which represents the unexplained variation in people’s starting cognitive scores, decreased steadily from Model 1 (SD = 3.12) to Model 3 (SD = 2.57), indicating reduced unexplained between-person differences after adding adult factors. The random slope variance also reflects the influence of these adult factors, decreasing from 0.61 in Model 1 to 0.52 in Model 2, indicating that adult social factors, particularly education, help explain the variability in cognitive decline over time. The slope variance then increased to 0.60 in the final model, suggesting a complex interplay between the adult-life factors and the individual rates of cognitive change. Finally, the model fit improved at each step, as indicated by the progressively lower AIC and BIC values.

Figure 1 illustrates the predicted cognitive trajectories from the final, fully adjusted multilevel model (Model 3), with 95% confidence intervals shown as shaded ribbons. Panels (A)-(F) consistently demonstrate the non-linear decline in cognitive function with age.

After controlling for adult social and health factors, the differences between some groups are subtle, visually confirming the sequential attenuation observed in Table 2. For instance, in panel (A) (Childhood financial status) and panel (F) (Relationship with father), the trajectories for the different groups lie very close together, with largely overlapping confidence intervals. This pattern aligns with Table 2, where the effects of these variables were substantially attenuated or became non-significant in the fully adjusted model.

In contrast, clear and persistent gaps remain for other early-life factors. As shown in panels (B) and (C), individuals whose mothers or fathers were in non-agricultural occupations had consistently higher cognitive function scores across the observed age range. Similarly, panel (D) indicates that those who never experienced childhood loneliness maintained a cognitive advantage across the lifespan. Finally, panel (E) demonstrates a clear dose-response association for relationship quality with mother, with “Very good or excellent” relationships associated with the higher predicted cognitive scores.

### Robustness check

Sensitivity analyses using multiple imputation produced results that were highly consistent with the primary analyses (Supplementary Table 3), confirming the associations between childhood financial status, psychosocial indicators, and later-life cognitive function. This consistency suggests that missing data did not materially influence the estimated associations.

To address potential attrition bias, we conducted a second sensitivity analysis, re-estimating our final model using only participants who completed all five waves (7,542 participants, 23,442 observations). The results were again highly consistent with our main findings (Supplementary Table 4). The key childhood indicators retained significance with very similar coefficients, indicating that the main findings are not substantially biased by participant attrition. Together, these checks support the robustness of the reported findings.

## Discussion

This study confirms the significant, long-term impact of childhood adversity on later-life cognitive function. Our findings indicate that although childhood economic and psychosocial disadvantages are strongly associated with lower baseline cognitive function, these associations are substantially attenuated once adult social and health factors are taken into account, suggesting that later-life circumstances partly explain the enduring influence of early disadvantage.

The association between poorer childhood financial status and lower cognitive scores was robust and consistent with recent global systematic reviews [16, 17]. These reviews synthesised evidence from studies using both subjective self-reports of childhood financial hardship and objective indicators such as parental education, occupation, and household amenities. Our sequential analysis, however, revealed that the effect of childhood financial status was almost entirely attenuated by adult factors, with the largest reduction on adding education. In contrast, parental occupation, notably the father’s, remained a significant and independent predictor even in the full model. This finding is particularly relevant in the unique historical context of this cohort in China. The childhoods of these participants unfolded during the Mao era, a period characterised by the implementation of the rigid hukou (household registration) system. However, this era was also defined by significant state-led efforts to expand education and healthcare access to rural populations. In this context, a father’s occupation as “Farming” was a state-defined status that, while linked to material scarcity, was also the primary target of social policies aimed at improving rural welfare and creating new pathways for social mobility [44]. This may explain why it has such a powerful and enduring “sensitive period” effect that persists even beyond an individual’s own adult achievements. This also supports other research linking non-agricultural occupations to better cognitive development, as they may offer greater educational opportunities [45] and less physically demanding work, leaving parents more energy to engage with their children [46].

Childhood psychosocial indicators also emerged as critical predictors. Not feeling lonely in childhood remained a strong protective factor, and its association with higher cognition persisted even in the full model. This supports the hypothesis that early psychosocial isolation may impair the development of cognitive reserve by limiting social interaction and stimulation [47]. Furthermore, a clear distinction emerged in parent-child relationships. The effect of the paternal relationship was fully attenuated, whereas the maternal relationship remained robust. This finding likely reflects the cohort’s traditional East Asian family structure, where men were primarily engaged in external work and women in domestic and caregiving roles [48]. In this context, mothers were the primary source of emotional security and early-life nurturing. This secure attachment is theorised to promote cognitive resilience, including better executive function and stress regulation, which can help children adapt to and cope with the psychological stressors of economic hardship, potentially mitigating its negative impact on cognitive development [49, 50].

Another key finding of this study was the explicit modelling of these life-course pathways, which confirmed our second hypothesis. Our results overwhelmingly show that education is the single most potent factor. The attenuation of all childhood factors after adding education to the models suggests that a primary mechanism by which childhood disadvantage harms later-life cognition is by restricting educational attainment. This is consistent with cognitive reserve theory, which posits that education builds resilience against cognitive decline [51]. Additionally, our results demonstrate that adult health status, specifically limitations in IADL and depression, critically influences cognitive ageing. Higher IADL needs were significantly associated with lower cognitive scores, supporting IADL function as an early predictor of cognitive status and suggesting that reduced functional independence may limit the cognitive engagement needed to maintain brain health [52]. Depression was also associated with lower cognitive function, potentially impairing cognition through stress-related biological pathways, including accelerated neurostructural ageing [53]. The results underscore the need for a holistic approach to preserving cognitive function that integrates mental health support and functional rehabilitation.

These findings must be interpreted within China’s unique socio-historical trajectory, which offers a notable contrast to life-course research in Western contexts. While studies in the US and Europe often frame childhood SES through stable markers of family class or parental education [54, 55], , the CHARLS cohort lived through a period of unprecedented transition from a state-organised socialist society, which prioritised the mobilisation of resources for the rural peasantry, to a rapid market economy. In Western nations, upward social mobility often provides broader opportunities for cognitive remediation through consistently available public resources [56]. In contrast, rapid development in China meant that many individuals achieved significant adult socioeconomic gains but remained shaped by a formative “sensitive period” of social restratification. While the hukou system created initial administrative boundaries, the socialist period’s emphasis on rural development provided unique institutional platforms that helped many children from farming backgrounds access education and health resources. Consequently, the persistent influence of early-life factors observed here aligns with the universal theory of social determinants of health, which suggests that the link between early-life circumstances and later-life outcomes is a consistent phenomenon observed across diverse sociopolitical models.

### Strengths and limitations

This study has several limitations. First, our childhood measures relied on retrospective self-reports, which may be subject to recall bias [57, 58]. This is a particular concern for subjective items like loneliness and parental relationships, and may be exacerbated in those already experiencing cognitive decline [57]. Second, the childhood finance item reflects perceived standing relative to the respondent’s local community rather than an objective measure of household wealth, so comparability may vary across communities with different overall affluence and misclassification across contexts is possible. Third, despite controlling for many covariates, there is always a risk of unmeasured confounding from factors like genetic predispositions or childhood nutrition [59]. Fourth, the realistic meaning of our results must be interpreted within their historical context. Participants were aged 45 and older in 2011, meaning their childhoods occurred decades ago. The applicability of our findings to contemporary childhood contexts may therefore be limited.

Significant strengths balance these limitations. We used a large, nationally representative longitudinal sample from CHARLS, enhancing the generalisability of our findings. Methodologically, this study is a notable improvement on prior work. We applied advanced multilevel growth curve models that included both random intercepts and random slopes. These models also captured non-linear change through quadratic age terms, which provides a more accurate and realistic model of cognitive ageing. Furthermore, this approach, estimated using maximum likelihood, provided a modern and robust method for handling the substantial missing data present in longitudinal ageing studies. Finally, we confirmed the stability of our findings by conducting a complete-case sensitivity analysis, which showed no significant changes in the results. This gives us confidence that our findings are robust and not simply an artefact of attrition bias.

### Implications

Our findings suggest a two-pronged approach to public health. First, the significance of early-life factors underscores the importance of primary prevention. The enduring effects of parental occupation and maternal relationships suggest that policies supporting agricultural families and integrating positive parenting support into routine child healthcare are effective long-term strategies for promoting cognitive health. Second, the decisive role of adult factors is a hopeful finding, as it suggests that it is never too late to intervene. The strong effect of education suggests that promoting adult education for individuals with low attainment could help build cognitive reserve. Finally, the strong association with IADL needs and depression means that routine screening for functional limitations and depressive symptoms in primary care may be an effective clinical strategy to disrupt the pathway from early-life adversity to cognitive decline.

## Conclusion

This study demonstrates that childhood disadvantages, both economic and psychosocial, have persistent, long-term effects on cognitive function in older Chinese adults. These effects are not just direct; our findings show that they are substantially explained through life-course pathways, particularly educational attainment and adult life physical and mental health. This highlights the need for a “whole-of-life” approach to cognitive health, suggesting that interventions to support children, as well as those aimed at promoting education and health in adulthood, are both critical strategies for reducing the future burden of cognitive decline in China.

## Supplementary Information

Supplementary Material 1.

## Data Availability

The dataset(s) supporting the conclusions of this article are available in the China Health and Retirement Longitudinal Study (CHARLS) repository. The data are publicly available to registered researchers at the following hyperlink: [https://charls.pku.edu.cn/en/](https:/charls.pku.edu.cn/en) .

## Abbreviations

ADL: Activities of Daily Living
AIC: Akaike Information Criterion
BIC: Bayesian Information Criterion
CESD-10: 10-item Center for Epidemiologic Studies Depression Scale
CHARLS: China Health and Retirement Longitudinal Study
CI: Confidence Interval
IADL: Instrumental Activities of Daily Living
SD: Standard Deviation
SE: Standard Error
SES: Socioeconomic Status
