# Supplementary material for: Associations of childhood economic and psychosocial conditions with later-life cognitive function: a longitudinal analysis of the China health and retirement longitudinal study (2011–2020)
Source: BMC Public Health. 2026 Feb 27;26:1107. doi: 10.1186/s12889-026-26826-2 (PMC13049817; doi:10.1186/s12889-026-26826-2)
Supplement: Supplementary file 1 — Supplementary Material 1. [file 12889_2026_26826_MOESM1_ESM.docx]

Appendix A. Supplementary data

# Supplementary Table 1 STROBE Statement: Checklist of items that should be included in reports of observational studies

|  | | Item No | Recommendation | Replace |
| --- | --- | --- | --- | --- |
| **Title and abstract** | | 1 | (*a*) Indicate the study’s design with a commonly used term in the title or the abstract | Title page |
|  |  |  | (*b*) Provide in the abstract an informative and balanced summary of what was done and what was found | Abstract (Page 2) |
| Introduction | | | |  |
| Background/rationale | | 2 | Explain the scientific background and rationale for the investigation being reported | Page 4-5 |
| Objectives | | 3 | State specific objectives, including any prespecified hypotheses | Page 5-6 |
| Methods | | | |  |
| Study design | | 4 | Present key elements of study design early in the paper | Page 6 |
| Setting | | 5 | Describe the setting, locations, and relevant dates, including periods of recruitment, exposure, follow-up, and data collection | Page 6 |
| Participants | | 6 | (*a*) *Cross-sectional study*—Give the eligibility criteria, and the sources and methods of selection of participants | Not applicable |
|  |  |  | (*b*) *Cohort study*—For matched studies, give matching criteria and number of exposed and unexposed  *Case-control study*—For matched studies, give matching criteria and the number of controls per case | Page 6 |
| Variables | | 7 | Clearly define all outcomes, exposures, predictors, potential confounders, and effect modifiers. Give diagnostic criteria, if applicable | Page 6-9 |
| Data sources/ measurement | | 8* | For each variable of interest, give sources of data and details of methods of assessment (measurement). Describe comparability of assessment methods if there is more than one group | Page 6-9 |
| Bias | | 9 | Describe any efforts to address potential sources of bias | Page 10 |
| Study size | | 10 | Explain how the study size was arrived at | Page 6 |
| Quantitative variables | | 11 | Explain how quantitative variables were handled in the analyses. If applicable, describe which groupings were chosen and why | Page 6-9 |
| Statistical methods | | 12 | (*a*) Describe all statistical methods, including those used to control for confounding | Statistical analysis  (Page 9-10) |
|  |  |  | (*b*) Describe any methods used to examine subgroups and interactions | Statistical analysis  (Page 9-10) |
|  |  |  | (*c*) Explain how missing data were addressed | Page 10 |
|  |  |  | (*d*) *Cross-sectional study*—If applicable, describe analytical methods taking account of sampling strategy | Not applicable |
|  |  |  | (*e*) Describe any sensitivity analyses | Page 10 |
| Results | | | |  |
| Participants | 13* | (a) Report numbers of individuals at each stage of study—eg numbers potentially eligible, examined for eligibility, confirmed eligible, included in the study, completing follow-up, and analysed | | Page 6 |
|  |  | (b) Give reasons for non-participation at each stage | | Page 6 |
|  |  | (c) Consider use of a flow diagram | | Not applicable |
| Descriptive data | 14* | (a) Give characteristics of study participants (eg demographic, clinical, social) and information on exposures and potential confounders | | Table 1 (Page 10-11) |
|  |  | (b) Indicate number of participants with missing data for each variable of interest | | Table 1 (Page 10-11) |
|  |  | (c) *Cohort study*—Summarise follow-up time (eg, average and total amount) | | Not applicable |
| Outcome data | 15* | *Cohort study*—Report numbers of outcome events or summary measures over time | | Page 10 |
|  |  | *Case-control study—*Report numbers in each exposure category, or summary measures of exposure | | Not applicable |
|  |  | *Cross-sectional study—*Report numbers of outcome events or summary measures | | Not applicable |
| Main results | 16 | (*a*) Give unadjusted estimates and, if applicable, confounder-adjusted estimates and their precision (eg, 95% confidence interval). Make clear which confounders were adjusted for and why they were included | | Table 2 (Page 13-14) |
|  |  | (*b*) Report category boundaries when continuous variables were categorized | | Table 2 (Page 13-14) |
|  |  | (*c*) If relevant, consider translating estimates of relative risk into absolute risk for a meaningful time period | | Not applicable |
| Other analyses | 17 | Report other analyses done—eg analyses of subgroups and interactions, and sensitivity analyses | | Figure 1 (Page 16), robustness check (Page 17) |
| Discussion | | | |  |
| Key results | 18 | Summarise key results with reference to study objectives | | Page 17-19 |
| Limitations | 19 | Discuss limitations of the study, taking into account sources of potential bias or imprecision. Discuss both direction and magnitude of any potential bias | | Page 19 |
| Interpretation | 20 | Give a cautious overall interpretation of results considering objectives, limitations, multiplicity of analyses, results from similar studies, and other relevant evidence | | Page 17-19 |
| Generalisability | 21 | Discuss the generalisability (external validity) of the study results | | Page 20 |
| Other information | | | |  |
| Funding | 22 | Give the source of funding and the role of the funders for the present study and, if applicable, for the original study on which the present article is based | | Not applicable |

*Give information separately for cases and controls in case-control studies and, if applicable, for exposed and unexposed groups in cohort and cross-sectional studies.

**Note:** An Explanation and Elaboration article discusses each checklist item and gives methodological background and published examples of transparent reporting. The STROBE checklist is best used in conjunction with this article (freely available on the Web sites of PLoS Medicine at http://www.plosmedicine.org/, Annals of Internal Medicine at http://www.annals.org/, and Epidemiology at http://www.epidem.com/). Information on the STROBE Initiative is available at www.strobe-statement.org.

# Supplementary Table 2 Multilevel growth curve models testing cross-level interactions between childhood economic and psychosocial conditions and age-related cognitive trajectories

| Variable | β (SE)  Main Effect | p-value  Main Effect |  | β (SE)  Age Interaction | p-value  Age Interaction | β (SE)  Age² Interaction | p-value  Age² Interaction |
| --- | --- | --- | --- | --- | --- | --- | --- |
| Childhood finance (ref: Same) |  |  |  |  |  |  |  |
| Worse | 2.093 (2.573) | 0.416 |  | -0.0750 (0.0838) | 0.37 | 0.000612 (0.000675) | 0.364 |
| Better | 1.673 (4.099) | 0.683 |  | -0.0474 (0.134) | 0.723 | 0.000300 (0.00107) | 0.779 |
| Mother’s occupation (ref: Farming) |  |  |  |  |  |  |  |
| Non-Agricultural | -3.872 (5.610) | 0.49 |  | 0.0866 (0.183) | 0.637 | -0.000218 (0.00148) | 0.883 |
| Father’s occupation (ref: Farming) |  |  |  |  |  |  |  |
| Non-Agricultural | 0.722 (3.593) | 0.841 |  | -0.00520 (0.118) | 0.965 | 0.0000905 (0.000958) | 0.925 |
| Childhood loneliness (ref: Lonely) |  |  |  |  |  |  |  |
| Never | -0.923 (2.984) | 0.757 |  | 0.0221 (0.0967) | 0.819 | -0.0000239 (0.000775) | 0.975 |
| Mother relationship (ref: Fair/Poor) |  |  |  |  |  |  |  |
| Good | 8.336 (5.489) | 0.129 |  | -0.267 (0.177) | 0.132 | 0.00214 (0.00142) | 0.131 |
| Very good/Excellent | 3.152 (4.593) | 0.493 |  | -0.116 (0.149) | 0.434 | 0.00115 (0.00119) | 0.337 |
| Father relationship (ref: Fair/Poor) |  |  |  |  |  |  |  |
| Good | -10.302 (5.265) | 0.05 |  | 0.357 (0.170) | 0.036 | -0.00296 (0.00136) | 0.029 |
| Very good/Excellent | -4.986 (4.444) | 0.262 |  | 0.187 (0.144) | 0.194 | -0.00164 (0.00115) | 0.155 |

*Note: β (SE) = Coefficient (Standard Error).

Supplementary Table 3 Sensitivity analysis using multiple imputation by chained equations (MICE)

| **Variable** | **Model 1** | | **Model 2** | | **Model 3** | |
| --- | --- | --- | --- | --- | --- | --- |
|  | **β (SE)** | **p-value** | **β (SE)** | **p-value** | **β (SE)** | **p-value** |
| Finance compared to other children (ref: Same) |  |  |  |  |  |  |
| Worse | -0.38 (0.07) | <0.001 | -0.22 (0.06) | <0.001 | -0.04 (0.06) | 0.491 |
| Better | 0.35 (0.11) | 0.002 | 0.03 (0.11) | 0.781 | -0.06 (0.10) | 0.568 |
| Mother's occupation (ref: Farming) |  |  |  |  |  |  |
| Non-Agricultural | 1.58 (0.15) | <0.001 | 0.48 (0.14) | 0.001 | 0.40 (0.14) | 0.003 |
| Father's occupation (ref: Farming) |  |  |  |  |  |  |
| Non-Agricultural | 1.25 (0.10) | <0.001 | 0.60 (0.09) | <0.001 | 0.49 (0.09) | <0.001 |
| Loneliness (ref: Yes) |  |  |  |  |  |  |
| Never | 0.72 (0.08) | <0.001 | 0.46 (0.07) | <0.001 | 0.24 (0.07) | 0.001 |
| Relationship with mother (ref: Fair/poor) |  |  |  |  |  |  |
| Good | 0.32 (0.13) | 0.017 | 0.23 (0.12) | 0.060 | 0.15 (0.12) | 0.196 |
| Very good/excellent | 0.46 (0.12) | <0.001 | 0.31 (0.11) | 0.006 | 0.22 (0.11) | 0.038 |
| Relationship with father (ref: Fair/poor) |  |  |  |  |  |  |
| Good | 0.22 (0.13) | 0.090 | 0.15 (0.12) | 0.210 | 0.12 (0.12) | 0.305 |
| Very good/excellent | 0.31 (0.12) | 0.008 | 0.21 (0.11) | 0.053 | 0.14 (0.10) | 0.172 |
| Age (linear) | -0.003 (0.03) | 0.939 | 0.03 (0.03) | 0.398 | 0.03 (0.03) | 0.287 |
| Age (quadratic) | -0.0006 (0.0002) | 0.027 | -0.0006 (0.0003) | 0.025 | -0.0005 (0.0003) | 0.052 |
| Sex (ref: Female) |  |  |  |  |  |  |
| Male | 1.02 (0.07) | <0.001 | 0.32 (0.06) | <0.001 | 0.10 (0.08) | 0.225 |
| Education (ref: Elementary or lower) |  |  |  |  |  |  |
| Secondary |  |  | 2.46 (0.07) | <0.001 | 2.20 (0.07) | <0.001 |
| High school or higher |  |  | 3.40 (0.16) | <0.001 | 2.94 (0.15) | <0.001 |
| Marital status (ref: Living alone) |  |  |  |  |  |  |
| Married/partnered |  |  | 0.90 (0.09) | <0.001 | 0.78 (0.09) | <0.001 |
| Living area (ref: Urban) |  |  |  |  |  |  |
| Rural |  |  | -1.12 (0.08) | <0.001 | -1.00 (0.08) | <0.001 |
| Smoking (ref: Never) |  |  |  |  |  |  |
| Yes |  |  |  |  | -0.09 (0.08) | 0.287 |
| Social activity (ref: No) |  |  |  |  |  |  |
| Yes |  |  |  |  | 0.72 (0.05) | <0.001 |
| Hypertension (ref: No) |  |  |  |  | -0.21 (0.06) | 0.001 |
| Diabetes (ref: No) |  |  |  |  | 0.005 (0.09) | 0.958 |
| Cancer (ref: No) |  |  |  |  | 0.24 (0.27) | 0.386 |
| Lung Disease (ref: No) |  |  |  |  | -0.19 (0.10) | 0.058 |
| Stroke (ref: No) |  |  |  |  | -0.05 (0.14) | 0.732 |
| Psychiatric (ref: No) |  |  |  |  | 0.16 (0.24) | 0.515 |
| Arthritis (ref: No) |  |  |  |  | -0.36 (0.06) | <0.001 |
| Asthma (ref: No) |  |  |  |  | -0.26 (0.13) | 0.051 |
| ADL needs help |  |  |  |  | -0.02 (0.04) | 0.584 |
| IADL needs help |  |  |  |  | -0.42 (0.04) | <0.001 |
| CESD-10 score |  |  |  |  | -0.09 (0.005) | <0.001 |

# *Note: β (SE) = Coefficient (Standard Error).

# Supplementary Table 4 Sensitivity analysis using complete-case participants to assess robustness against participant attrition (N = 7,542; observations = 23,442)

| Variable | β (SE) | p-value |
| --- | --- | --- |
| Finance compared to other children (ref: Same) |  |  |
| Worse | -0.18 (0.08) | 0.026 |
| Better | 0.02 (0.14) | 0.876 |
| Mother's occupation (ref: Farming) |  |  |
| Non-Agricultural | 0.59 (0.20) | 0.003 |
| Father's occupation (ref: Farming) |  |  |
| Non-Agricultural | 0.79 (0.12) | <0.001 |
| Loneliness (ref: Yes) |  |  |
| Never | 0.31 (0.10) | 0.001 |
| Relationship with mother (ref: Fair/poor) |  |  |
| Good | 0.15 (0.17) | 0.395 |
| Very good/excellent | 0.34 (0.15) | 0.021 |
| Relationship with father (ref: Fair/poor) |  |  |
| Good | 0.29 (0.16) | 0.081 |
| Very good/excellent | 0.21 (0.14) | 0.129 |
| Age (linear) | 0.40 (0.05) | <0.001 |
| Age (quadratic) | -0.004 (0.000) | <0.001 |
| Sex (ref: Female) |  |  |
| Male | 0.78 (0.11) | <0.001 |
| Education (ref: Elementary or lower) |  |  |
| Secondary | 2.75 (0.09) | <0.001 |
| High school or higher | 3.62 (0.22) | <0.001 |
| Marital status (ref: Living alone) |  |  |
| Married/partnered | 0.40 (0.11) | <0.001 |
| Living area (ref: Urban) |  |  |
| Rural | -0.68 (0.10) | <0.001 |
| Smoking (ref: Never) |  |  |
| Yes | -0.22 (0.10) | 0.027 |
| Social activity (ref: No) |  |  |
| Yes | 0.63 (0.05) | <0.001 |
| Hypertension (ref: No) | 0.07 (0.07) | 0.307 |
| Diabetes (ref: No) | 0.23 (0.11) | 0.039 |
| Cancer (ref: No) | 0.52 (0.28) | 0.058 |
| Lung Disease (ref: No) | 0.03 (0.10) | 0.795 |
| Stroke (ref: No) | 0.02 (0.17) | 0.917 |
| Psychiatric (ref: No) | -0.26 (0.24) | 0.284 |
| Arthritis (ref: No) | -0.07 (0.07) | 0.3 |
| Asthma (ref: No) | 0.02 (0.17) | 0.924 |
| ADL needs help | -0.01 (0.04) | 0.777 |
| IADL needs help | -0.32 (0.04) | <0.001 |
| CESD-10 score | -0.09 (0.01) | <0.001 |

*Note: β (SE) = Coefficient (Standard Error).
